# “Three-Bullets” Loaded Mesoporous Silica Nanoparticles for Combined Photo/Chemotherapy †

**DOI:** 10.3390/nano9060823

**Published:** 2019-05-31

**Authors:** André Luiz Tessaro, Aurore Fraix, Ana Claudia Pedrozo da Silva, Elena Gazzano, Chiara Riganti, Salvatore Sortino

**Affiliations:** 1 Laboratory of Photochemistry, Department of Drug Sciences, University of Catania, 95125 Catania, Italy; fraix@unict.it; 2 Department of Chemistry, Federal University of Technology, Paraná, R. Marcílio Dias, 635, Jardim Paraíso, Apucarana 86812-460, Paraná, Brazil; 3 Department of Chemistry, Universidade Estadual de Maringá, Av. Colombo, 5.790, Maringá 87.020-900, Paraná, Brazil; anapedrozo1@gmail.com; 4 Department of Oncology, University of Torino, Via Santena 5/bis, I-10126 Torino, Italy; elena.gazzano@unito.it (E.G.); chiara.riganti@unito.it (C.R.)

**Keywords:** multimodal therapy, singlet oxygen, doxorubicin, nitric oxide

## Abstract

This contribution reports the design, preparation, photophysical and photochemical characterization, as well as a preliminary biological evaluation of mesoporous silica nanoparticles (MSNs) covalently integrating a nitric oxide (NO) photodonor (NOPD) and a singlet oxygen (^1^O_2_) photosensitizer (PS) and encapsulating the anticancer doxorubicin (DOX) in a noncovalent fashion. These MSNs bind the NOPD mainly in their inner part and the PS in their outer part in order to judiciously exploit the different diffusion radius of the cytotoxic NO and ^1^O_2_. Furthermore this silica nanoconstruct has been devised in such a way to permit the selective excitation of the NOPD and the PS with light sources of different energy in the visible window. We demonstrate that the individual photochemical performances of the photoactive components of the MSNs are not mutually affected, and remain unaltered even in the presence of DOX. As a result, the complete nanoconstruct is able to deliver NO and ^1^O_2_ under blue and green light, respectively, and to release DOX under physiological conditions. Preliminary biological results performed using A375 cancer cells show a good tolerability of the functionalized MSNs in the dark and a potentiated activity of DOX upon irradiation, due to the effect of the NO photoreleased.

## 1. Introduction

Multimodal cancer therapy involves the use of two or more treatment modalities with the aim to attack tumors on different sides by acting either on a single oncogenic pathway through different mechanisms or across parallel pathways without amplification of side effects [1,2,3,4]. In this frame, the combination of conventional chemotherapeutics with light-activated therapeutic treatments is a very appealing approach to potentiate the therapeutic outcome through anticancer synergistic/additive effects [5]. Light represents in fact as suitable and minimally invasive tool that permits the introduction of therapeutic species in biological environments with superb spatiotemporal control [6]. Among the light-activatable therapeutic treatments, photodynamic therapy (PDT) is so far one of the most promising to combat cancer diseases [7]. This treatment modality mainly exploits the cytotoxic effects of the highly reactive singlet oxygen (^1^O_2_), catalytically generated by energy transfer between the long-lived excited triplet state of a photosensitizer (PS) and the nearby molecular oxygen [8].

Another emerging light-activated approach is based on the photoregulated release of nitric oxide (NO) through the use of NO photodonors (NOPD) [9,10,11]. Although still confined to the research area, this NO-based PDT, namely NOPDT, has come to the limelight in recent years and holds very promising features in cancer treatment [12]. In fact, apart from playing multiple roles in the bioregulation of a broad array of physiological processes [13], NO has also proven to be an effective anticancer agent. If generated in an appropriate concentration range [14,15] this diatomic free radical can act not only as a cytotoxic agent [16], but also as inhibitor of the ATP binding cassette (ABC) transporters responsible of the efflux of chemotherapeutics and of the induction of multidrug resistance (MDR) in cancer cells [17,18]. In contrast to the photocatalytic mechanism leading to ^1^O_2_ in PDT, the working principle of NOPDT exploits the excitation light to uncage NO leading, of course, to a consumption of the NOPD [9,10,11]. Noteworthy, both ^1^O_2_ and NO are multitarget species that have not a reduced efficacy in MDR cells due to their short lifetimes, and confine their action to short distances from the production site inside the cells (<20 nm for ^1^O_2_ and <200 µm for NO), reducing systemic toxicity issues common to many conventional drugs. Besides, since NO photorelease is independent of O_2_ availability, it may successfully complement PDT at the onset of hypoxic conditions, typical for some tumors, where PDT may fail [19].

The significant breakthroughs in nanomedicine permit nowadays the development of multifunctional platforms in which more therapeutic agents are entrapped in a single nanocarrier. This offers an unprecedented opportunity to devise a better scheme for precise and controlled delivery of multiple therapeutics to the same area of the body at a predefined extra/intracellular level [20,21,22,23]. In this context, nanoplatforms combining PDT and NOPDT are opening new horizons towards more effective and less invasive cancer treatments entirely based on “nonconventional” chemotherapeutics [24]. Besides, an increasing large number of nanoconstructs in which PDT has been combined with chemotherapy in the management of several cancer types has been reported in literature [5]. In contrast, only recently NOPDT has been successfully used in combination with chemotherapeutics [25]. Based on this scenario, the achievement of a trimodal nanoplatform able to generate ^1^O_2_ and NO and, simultaneously, to deliver a conventional chemotherapeutic is a very challenging objective to pursue. Mesoporous silica nanoparticles (MSNs) are very suitable scaffolds to this end due to their high loading capacity, ease of surface functionalization, good biocompatibility, and satisfactory light transparency [26,27,28,29,30]. Moreover these inorganic scaffolds have been extensively used as excellent carrier for (i) conventional chemotherapeutics to reverse MDR [31], (ii) PDT agents alone and in combinations with chemotherapeutics [32,33,34], and (iii) spontaneous NO donors [35] and NOPD [36].

In this manuscript, we report the first example of engineered MSNs able to deliver “three bullets” for potential anticancer therapy. These MSNs covalently integrate in their molecular scaffold erythrosine and a nitroaniline derivative as suitable PS and NOPD, respectively, and encapsulate the chemotherapeutic doxorubicin (DOX) in a noncovalent fashion. We demonstrate that the photochemical properties of the PS and NOPD are preserved in an excellent manner in the MSNs containing all active components. As a result, the spontaneous release of DOX under physiological conditions can be combined with the photoregulated generation of ^1^O_2_, NO, or both by using light stimuli of appropriate energy in the visible light range.

## 2. Materials and Methods

### 2.1. Materials

All reagents (Sigma-Aldrich, Saint Louis, MO, USA) were of high commercial grade and were used without further purification. All solvents used (from Carlo Erba, Val de Reuil, France) were spectrophotometric grade. The free NOPD was synthesized according to our previously published procedure [37].

### 2.2. Synthetic Procedures

#### 2.2.1. Synthesis of Amino-Modified MSNs (MSNs-NH_2_)

MSNs-NH_2_ were prepared according to literature procedures using cetyltrimethylammonium bromide (CTAB) as template [38]. Briefly, CTAB (1.9 mmoL) was dissolved in 340 mL of water followed by the addition of 2.45 mL of NaOH (2.0 mol L^−1^). The temperature was adjusted at 80 °C and tetraethoxy silane (TEOS), 3.5 mL, 18.1 mmoL, and 3-aminopropyl triethoxy silane (APTES), (0.43 mL, 2.04 mmol) were simultaneously added dropwise to the solution during 4 min. The mixture was stirred at 80 °C for 2 h to give rise to white precipitate. The solid product was filtered, washed with deionized water and ethanol, and dried at 60 °C for 24 h.

#### 2.2.2. Synthesis of the PS-Modified MSNs (PS-MSNs)

500 mg of MSNs-NH_2_ were added in 15 mL of DMF and sonicated (90 s, 42 KHz). Then PS erythrosine (27 mg, 1 eq), O-(7-Azabenzotriazol-1-yl)-1,1,3,3-tetramethyl uronium hexafluorophosphate (11.1 mg, 1 eq) and N,N-di-isopropiletilamine (10 µL, 2 eq) were added to the suspension. The mixture was stirred at room temperature for 24 h under dark conditions. The solid product was filtered, washed with DMF (4 portions of 5 mL) and dried at 60 °C for 24 h. PS-MSNs were prepared with the nominal concentration of ca. 0.03% (*w*/*w*) of PS. The real concentration of the PS was indirectly determined by UV–Vis spectrum of the eluate (post washing) using ε_543_ = 112.000 M^−1^ cm^−1^ in DMF.

#### 2.2.3. Synthesis of NOPD-Modified PS-MSNs (PS-MSNs-NOPD)

One-hundred milligrams of PS-MSNs was added in 10 mL of DMSO and sonicated (90 s, 50 KHz). Then cesium carbonate (10 mg, 0.2 eq) and 4-chloro-1-nitro-2(trifluoromethyl) benzene (30 µL, 2 eq) were added and refluxed overnight. The solid product was filtered and washed with ethyl acetate (to remove the unbounded 4-chloro-1-nitro-2(trifluoromethyl) benzene) and water (to remove the excess of cesium carbonate) and dried under vacuum to obtain PS-MSNs-NOPD. PS-MSNs-NOPD were prepared with the nominal concentration of ca. 0.4% (*w*/*w*) of NOPD. The real concentration of the NOPD was indirectly determined by UV–Vis spectrum of the eluate (post washing) using ε_400_ = 10.000 M^−1^ cm^−1^ in water.

#### 2.2.4. Stability of the PS-MSNs-NOPD

Representative samples of PS-MSNs-NOPD (1 mg mL^−1^) were either sonicated for 1 h or stirred at 70 °C for 2 h in the dark. Afterwards the samples were centrifuged for 25 min and the supernatant was collected and analyzed by UV-VIS absorption. The absence of any relevant absorption between 250 and 800 nm, suggested that no leaching of the chromogenic units from the MSN scaffold occurs.

### 2.3. Instrumentation

Transmission electron microscopy (TEM) experiments were performed with a TEM JEOL JEM-2010 (Pleasanton, CA, USA) using the bright field in conventional parallel beam (CTEM) mode (BF).

The X-ray diffraction analysis were done at the Complexo de Apoio à Pesquisa (COMCAP/UEM) in Bruke diffractometer model D8 Advance with radiation Cu-Kα (λ = 1.54062 Å). The scanning was done from 1 to 10° of 2θ with 0.45°/min.

UV-Vis spectra absorption and fluorescence emission spectra were recorded with a JascoV-560 spectrophotometer (Easton, MD, USA) and a Spex Fluorolog-2 (mod. F-111) spectrofluorimeter, respectively, in air-equilibrated solutions, using either quartz cells with a path length of 1 cm. Fluorescence lifetimes were recorded with the same fluorimeter equipped with a TCSPC Triple Illuminator (Kyoto, Japan). The samples were irradiated by a pulsed diode excitation source (Nanoled) at 455 nm and the decays were monitored at 500 nm. The system allowed measurement of fluorescence lifetimes from 200 ps. The multiexponential fit of the fluorescence decay was obtained using the following equation.

I(t) = ∑α_I_ exp(^−t/τi^)

Absorption spectral changes were monitored by irradiating the sample in a thermostated quartz cell (1 cm path length, 3 mL capacity) under gentle stirring, using a continuum laser with λ_exc_ = 405 nm and λ_exc_ = 532 nm (ca. 100 mW) and having a beam diameter of ca. 1.5 mm for the excitation of the NOPD and the PS, respectively.

Direct monitoring of NO release in solution was performed by amperometric detection (World Precision Instruments, Sarasota, FL, USA), with a ISO-NO meter, equipped with a data acquisition system, and based on direct amperometric detection of NO with short response time (<5 s) and sensitivity range 1 nM to 20 µM. The analogue signal was digitalized with a four-channel recording system and transferred to a PC. The sensor was accurately calibrated by mixing standard solutions of NaNO_2_ with 0.1 M H_2_SO_4_ and 0.1 M KI according to the following reaction.

4H^+^ + 2I^−^ + 2NO_2_^−^ → 2H_2_O + 2NO + I_2_

Irradiation was performed in a thermostated quartz cell (1 cm path length, 3 mL capacity) using the continuum laser with λ_exc_ = 405 nm. NO measurements were carried out under stirring with the electrode positioned outside the light path in order to avoid NO signal artifacts due to photoelectric interference on the ISO-NO electrode.

### 2.4. Chemical Detection of NO

NO release was also measured indirectly by means of the well-known, highly sensitive (detection limit on the order of the picomoles) fluorimetric bioassay of Misko et al. [39] based on the ring closure of the nonfluorescent 2,3-diaminonaphthalene (DAN) with nitrite to form the highly fluorescent product 2,3-diaminonaphthotriazole (DANT). Aliquots of 2 mL of aqueous suspension of the desired MSNs were irradiated or kept in the dark. Afterwards, the samples were centrifuged by 25 min and 1 mL of the supernatant was collected. One-hundred microliters of DAN solution (DAN 0.30 M in 0.62 M HCl) was added to 1 mL of the supernatant and solutions were stirred for 20 min at room temperature. Three-hundred microliters of NaOH 3 M was added in previous solution and stirred for 20 min at room temperature. The resultant solution was put into the fluorescent cuvette and the fluorescence was recorded at λ_exc_ = 360 nm.

### 2.5. Chemical Detection of Singlet Oxygen

The release of ^1^O_2_ by the MSNs was determined by indirect measurements using the well-established traps of ^1^O_2_ such as 1,3-diphenylisobenzofuran (DPBF) [40] and *p*-nitrosodimethylaniline/histidine (RNO/His) [41] in ethanol and phosphate buffer (pH = 7.4), respectively. The reaction between the scavengers and ^1^O_2_ can be followed by the UV-Vis absorption by monitoring the bleaching of the traps at 400 nm and 440 nm in the case of DPBF and RNO/His, respectively. The free PS in ethanol or in PBS was used as standard and the ^1^O_2_ delivery efficiency (*η_∆_*) can be calculated by the equation the following equation [42].

$$\eta_{\Delta particle}\;=\;\Phi_{ERY}\frac{t_{ERY}}{t_{particle}}$$

Briefly, the scavenger is transferred into a suspension of the nanoparticle (1 mg mL^−1^) or a solution containing the PS and, under constantly stirring, irradiated at time intervals. In order to avoid intense scattering the spectrum was registered after the deposition of the particles on the bottom of cuvette. The absorptions of both solutions were normalized at 532 nm assuming that the real absorption of the PS bounded in the MSNs can be calculated by measuring the absorption spectrum of a suspension of PS-MSNs and subtracting the baseline scattering by multiple point level baseline correction.

### 2.6. Loading and Release of DOX

Four-and-a-half micrograms of PS-MSNs-NOPD was mixed with 4.0 mL of PBS (pH = 7.4) containing DOX (220 µM). After stirring for 24 h, in the dark and at room temperature, the DOX-loaded PS-MSNs-NOPD (PS-MSNs-NOPD/DOX) were collected by centrifugation. The samples were washed with PBS and the content of the DOX was determined by comparison between the absorbance of the supernatant and the original solution at 482 nm (ε_482_ = 10.000 M^−1^ cm^−1^). A dispersion containing 1 mg of PS-MSNs-NOPD/DOX in 2.0 mL of PBS (pH 7.4) was incubated at 37 °C, in the dark under continuous stirring. At specified time, 100 µL of the supernatant was taken out after centrifugation (15,000× *g* rpm for 2 min) and an equal volume of fresh PBS was added to keep the sink condition. The concentration was determined by reading the fluorescence emission of DOX at 594 nm.

### 2.7. Biological Assays

#### 2.7.1. Cells Characterization

Human melanoma A375 cells (ATCC, Manassas, VA, USA) were maintained in DMEM medium supplemented with 10% *v*/*v* fetal bovine serum, 1% *v*/*v* penicillin-streptomycin, 1% *v*/*v* l-glutamine. Cells were maintained in a humidified atmosphere at 37 °C, 5% CO_2_. To measure the expression of ABC transporters, 1 × 10^6^ cells were rinsed and fixed with 2% *w*/*v* paraformaldehyde (PFA) for 2 min, washed three times with PBS and stained with anti-P-glycoprotein (Pgp/ABCB1) (Kamiya, Hamamatsu City, Japan), anti-MDR-related protein 1 (MRP1/ABCC1) (Abcam, Cambridge, UK) and anti-breast cancer resistance protein (BCRP/ABCG2) (SantaCruz Biotechnology Inc., Santa Cruz, CA, USA) antibodies for 1 h on ice, followed by an AlexaFluor 488-conjugated secondary antibody (Millipore, Billerica, MA, USA) for 30 min. One-hundred-thousand cells were analyzed with EasyCyte Guava™ flow cytometer (Millipore), equipped with the InCyte software (Millipore). Control experiments included incubation with non-immune isotype antibody.

#### 2.7.2. Viability Assays

Cells were incubated for 4 h with the different MSNs samples (1 mg mL^−1^). Cells were maintained in the dark or irradiated at room temperature for 30 min with a blue LED at 400 nm with an irradiance of 1.75 mW/cm^2^; irradiance was measured with a Delta Ohm HD2302.0 lightmeter equipped with a Delta Ohm LP4771RAD light probe. Twenty-four hours after the irradiation, cells were stained with 5% *w*/*v* crystal violet aqueous solution in 66% *v*/*v* methanol, washed twice with water, solubilized with 10% *v*/*v* acetic acid. The absorbance was read at 570 nm, using a Packard EL340 microplate reader (Bio-Tek Instruments, Winooski, VT, USA). The absorbance of untreated cells was considered as 100% viability; the results were expressed as percentage of viable cells versus untreated cells.

#### 2.7.3. Statistical Analysis

Data are provided as means ± SD. The results were analyzed by a one-way ANOVA and Tukey’s test. *p* < 0.05 was considered significant.

## 3. Results and Discussion

**Scheme 1:**
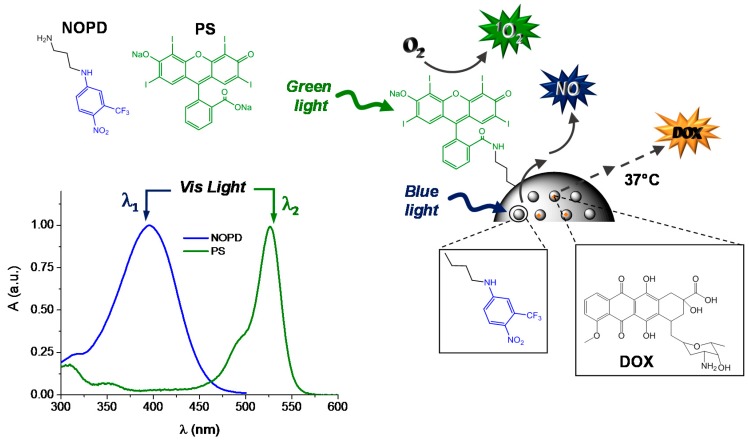
Schematic for the “three-bullets” PS-MSNs-NOPD/DOX and their working principle. The molecular structures of the free photosensitizer (PS) and the nitric oxide photodonor (NOPD) and their normalized absorption spectra in PBS (10 mM, pH 7.4) are also shown.

Scheme 1 illustrates the “three-bullets” MSNs with their working principle, and the rationale behind their design is described in the following.

As outlined in the introduction, ^1^O_2_ and NO have diffusion radius of <20 nm and <200 µm, respectively, due to their short lifetimes: ca. 3 µs for ^1^O_2_ [43] and ca. 5 s for NO [12]. Taking this into account we designed the MSNs in order to integrate mainly the PS in the outer part of the scaffold and the NOPD in the inner pores. This strategy avoids that ^1^O_2_ decays before to get out from the scaffold. On the other hand, despite being photogenerated in the core of the scaffold NO can diffuse out due to its longer lifetime. To this end, initially we synthesized the PS-MSNs before to eliminate the CTAB template (see experimental) from the amino terminated MSNs-NH_2_. In such a way, most of the condensation reaction between MSNs-NH_2_ and the free PS takes place at the scaffold surface. Afterwards, most of the CTAB was eliminated by the pores allowing the covalent integration of the NOPD mainly therein, according with the procedure described in the experimental.

An important issue to be addressed in the fabrication of phototherapeutic systems aimed at exploiting the effects of ^1^O_2_ and NO, regards the relative concentration of these species generated upon light excitation. As outlined above, ^1^O_2_ is produced through a photocatalytic process that, in principle, does not consume the PS, whereas photogeneration of NO occurs through a neat photochemical reaction with consequent consumption of the NOPD. As a consequence, the regulation of the reservoir of NO with respect to ^1^O_2_ is a critical point to be considered in view of an effective bimodal photodynamic action. This can be accomplished by (i) using a larger amount of the NOPD with respect to the PS, (ii) using PS and NOPD preferentially absorbing in different spectral regions, or (iii) combining both conditions. The appropriate synthetic protocol for the anchoring of the PS and NOPD permits to fulfill the first condition due to the regulation of the relative concentrations of distinct subsets of chromogenic units integrated within the very same scaffold. For such a reason, the amount of NOPD bounded to the MSNs was much larger than the PS (0.4% vs. 0.03%). Besides, the appropriate selection of the excitation wavelengths fulfills the second condition. On this basis, one can realize that the choice of erythrosine as PS and the nitroaniline derivative as NOPD is, of course, not casual. In fact, as shown in the spectra reported in Scheme 1, these two photoprecursors absorb in distinct regions of the visible spectral range and therefore can be selectively or simultaneously excited by using blue light, green light or both.

**Figure 1:**
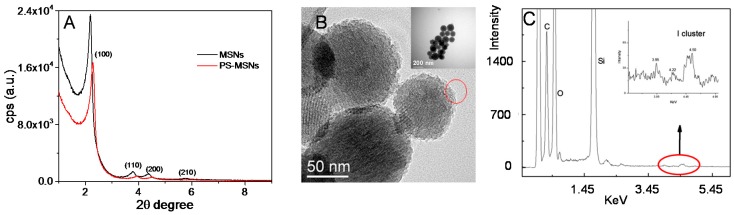
(**A**) X-ray powder diffraction (XRD) patterns of mesoporous silica nanoparticles (MSNs) (black line) and PS-MSNs (red line). (**B**) Representative TEM images and (**C**) EDX analysis of the PS-MSNs. The inset B shows a homogeneous distribution of the nanoparticles and the inset of C shows a zoom in the region of the iodide cluster.

PS-MSNs were characterized by X-ray powder diffraction (XRD), TEM, and energy-dispersive X-ray spectroscopy (EDX) analysis in order to verify their structural parameters and particle size. Figure 1A shows the diffractograms for the original material (MSNs) as well for the functionalized PS-MSNs. The typical reflections at 100, 110, 200, and 210 (not well resolved) confirm the production of an ordered hexagonal network material. The covalent addition of the PS did not affect the structural integrity of the material. The shift to higher values of 2θ can indicate a contraction of the porous due to the condensation of the sylanol groups. TEM microscopy (Figure 1B) shows particles with reasonable homogeneity regarding size and shape having an average size around 100 nm. EDX analysis (Figure 1C) confirms the anchoring of the PS by the signal of iodide in the external surface.

**Figure 2:**
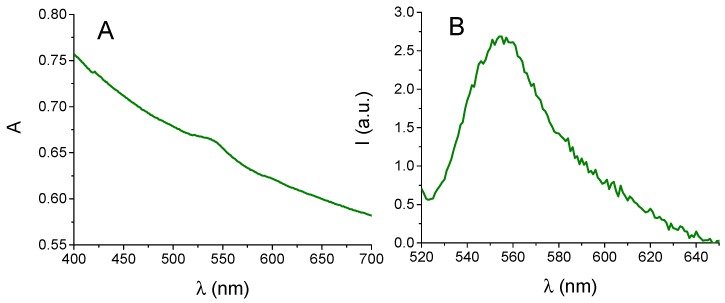
(**A**) Absorption and (**B**) fluorescence emission spectra (λ_exc_ = 510 nm) of PS-MSNs suspension (1 mg mL^−1^) in PBS (pH 7.4, 10 mM).

The binding of the PS to the MSNs was further confirmed by the absorption and fluorescence emission spectra of the PS-MSNs (Figure 2). The aqueous suspension of PS-MSNs shows the characteristic absorption of the PS at 537 nm, slightly red-shifted with respect to that of unbounded PS (see absorption spectrum in Scheme 1), and its typical fluorescence emission in the green region with maximum at ca. 550 nm.

**Figure 3:**
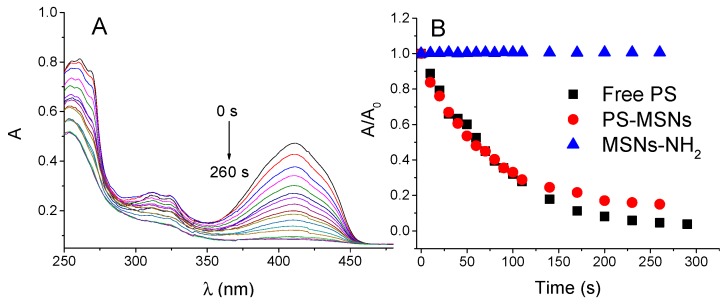
(**A**) Absorption spectral changes of DPBF (25 µM) observed upon irradiation at 532 nm (50 mW) in the presence of PS-MSNs suspension (1 mg mL^−1^) in ethanol. (**B**) A/A0 ratio at 411 nm of DPBF (25 µM) observed upon irradiation at 532 nm (50 mW) in the presence of free PS and suspensions of PS-MSNs (1 mg mL^−1^) and MSN-NH_2_ (1 mg mL^−1^) in ethanol.

The ^1^O_2_ photogeneration of the PS-MSNs was preliminary demonstrated by using DPBF, a well-known trap for this reactive oxygen species (see experimental). Figure 3 shows that the bleaching of the typical absorption band of the DPBF at 411 nm is observed only in the case of the MSNs functionalized with the PS. Furthermore, a control experiment performed with an optically matched solution of the free PS clearly show that the bleaching kinetic of the PS is not affected upon its covalent binding with the silica scaffold, fully supporting its localization at the MSNs surface.

**Figure 4:**
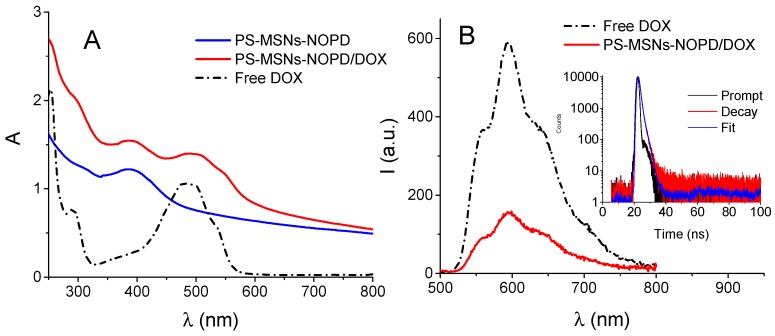
(**A**) Absorption spectra of free doxorubicin (DOX) and PS-MSNs-NOPD and PS-MSNs-NOPD/DOX suspensions (1 mg mL^−1^) in PBS (pH 7.4, 10 mM). (**B**) Fluorescence emission spectra (λ_exc_ = 490 nm) of free DOX and PS-MSNs-NOPD/DOX suspension (1 mg mL^−1^) in PBS (pH 7.4, 10 mM). The two samples are optically matched at the excitation wavelength. The inset shows the fluorescence decay and the related biexponential fitting for the PS-MSNs-NOPD/DOX suspension (λ_exc_ = 455 nm, λ_em_ = 590 nm).

The nitroaniline NOPD previously developed in our group [44] was covalently integrated in one-step into the PS-MSNs to give PS-MSNs-NOPD by reaction with the colorless chloro-nitroderivative (see experimental). Figure 4A shows unambiguous evidence for the successful synthesis of the PS-MSNs-NOPD. The absorption is in fact dominated by the typical charge transfer band of the nitroaniline chromophore in the blue region [44]. Note that, this absorption is ca. 10 nm blue-shifted if compared to that observed for the free NOPD in aqueous medium (see spectrum in Scheme 1, for sake of comparison), probably as a result of the different environment experienced by this chromogenic unit into the pore of the silica scaffold. This finding is in excellent agreement to what we recently observed for the same NOPD anchored on similar MSNs [36]. Note that, the introduction of the NOPD functionality into the nanoscaffold did not affect the particles size whose diameter was still of ~100 nm. 

The covalent binding of the NOPD mainly in the inner part of the MSNs does not preclude their capability to encapsulate in a noncovalent fashion additional therapeutic compounds. As a proof of principle, we selected Doxorubicin (DOX) as a suitable chemotherapeutic. This drug has a wide spectrum of activity in clinical application but it has to face the development of MDR in cancer cells [45]. The ABC transporters Pgp/ABCB1, MRP1/ABCC1 and BCRP/ABCG2 are the main transporters effluxing DOX from tumor cells [46]. The spectrum shown in Figure 4A clearly shows the effective encapsulation of DOX within the PS-MSNs-NOPD. In fact, the absorption band of the NOPD is accompanied by the characteristic band of the DOX with maximum at ca. 488 nm (see spectrum of the free DOX in Figure 4, for sake of comparison). DOX encapsulation was further confirmed by the appearance of its typical fluorescence emission in the red region (Figure 4B). Due to the scattering of the silica scaffold, the emission efficiency of the encapsulated DOX can be only estimated, resulting in ~70% smaller than the free drug. However, the biexponential decay observed for the PS-MSNs-NOPD/DOX (see inset Figure 4B) gave lifetimes of 0.54 and 2.1 ns, which were basically the same to those observed for the free DOX. These findings probably suggest the presence of some either non-emissive or scarcely emissive aggregates of DOX (these latter with lifetime below our time-resolution window) within the pores of the silica scaffold. The percentage of the DOX incorporated was ca. 85% corresponding to 90 µg of DOX per mg of MSNs

**Figure 5:**
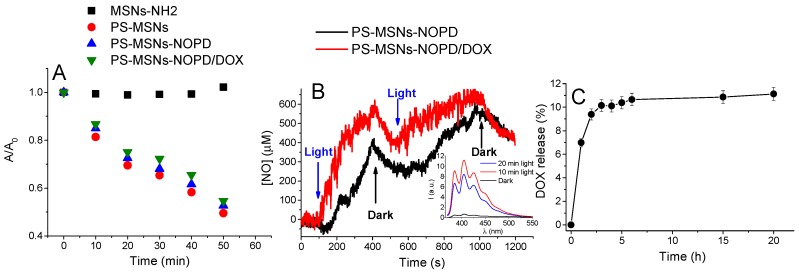
(**A**) A/A0 ratio at 440 nm of *p*-nitrosodimethylaniline/histidine (RNO) (17 µM) observed upon irradiation at 532 nm (50 mW) in the presence of different suspensions (1 mg mL^−1^) of MSNs in PBS (pH 7.4, 10 mM). (**B**) NO release profile observed upon irradiation at 405 nm (100 mW) of different suspensions (1 mg mL^−1^) of MSNs in PBS (pH 7.4, 10 mM). The inset shows the indirect e NO fluorimetric detection (λ_exc_ = 365 nm) (see experimental) observed for PS-MSNs-NOPD suspension (1 mg ml^−1^) in the dark and after 10 and 20 min irradiation at 405 nm. (**C**) DOX release observed for PS-MSNs-NOPD/DOX suspension (1 mg mL^−1^) in PBS (pH 7.4, 10 mM) at 37 °C.

In order to demonstrate the validity of the “three bullets” MSNs, we performed ^1^O_2_ and NO measurements under green and blue light stimuli, respectively, and DOX release experiments at 37 °C. In this case, we used *p*-nitrosodimethylaniline/histidine (RNO/His), a well-known ^1^O_2_ scavenger in aqueous PBS. In fact, in contrast to the previously used DPBF, RNO absorption maximum is at ca. 440 nm and thus is less affected by the absorption of the NOPD unit. Figure 5A shows the evolution of the absorbance bleaching for the unfunctionalized MSNs, those integrating only PS, both PS and NOPD, and the complete nanoplatform also loading DOX. The bleaching monitored at 440 nm upon 532 nm light excitation was observed in all samples containing the PS and provides a clear cut evidence for the delivery of ^1^O_2_, in contrast to the result observed for the unfunctionalized MSNs. Interestingly, the efficiency for the ^1^O_2_ observed in the case of the complete system PS-MSNs-NOPD/DOX was basically comparable with the samples not loaded with DOX and not containing the NOPD, suggesting that the copresence of the NOPD and DOX does not influence the photophysical properties of the bound PS.

The NO photorelease properties of PS-MSNs-NOPD/DOX were unambiguously demonstrated by direct real-time monitoring using an ultrasensitive NO electrode, which directly detects NO with nM concentration sensitivity employing an amperometric technique. The results illustrated in Figure 5B provide evidence that the nanoconstruct is stable in the dark, whereas it releases NO upon blue light excitation at 405 nm, stops as the light is turned off, and restarts again upon illumination. Also in this case, a comparison with the sample not loaded with DOX, confirmed that the efficiency of the NO photorelease is basically not affected by the presence of the chemodrug. NO photorelease was further proved by the indirect DAN assay (see experimental), one of the most sensitive and selective fluorescence-based methods for NO detection as nitrite (see experimental). As evident from the inset of Figure 5B, the significant fluorescence for of the nitrite probe was observed only in the case of the irradiated samples.

DOX release from the nanoparticles was performed under sink conditions at 37 °C in PBS at pH 7.4. Figure 5C illustrates the releasing profile of the DOX monitored for 24 h. After 3 h, ca. 10% of DOX was released suggesting that the presence of the NOPD and the PS in the silica scaffold do not change the typical release profile of DOX. Note that the amount of released chemodrug is comparable with that observed for DOX in similar MSN scaffolds [34].

**Figure 6:**
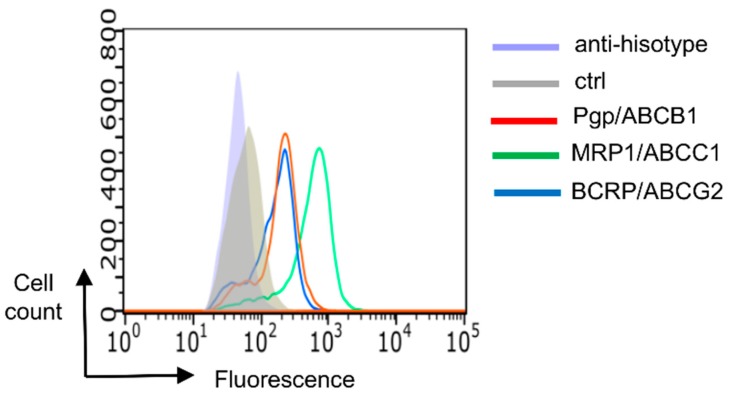
Representative histograms of Pgp/ABCB1, MRP1/ABCC1, and BCRP/ABCG2 surface levels in A375 cells, measured by flow cytometry in duplicate. The figure is representative of 1 out of 3 experiments with similar results.

As outlined in the introductory part, NO may play a double role when combined with chemotherapeutics. It can increase the therapeutic effectiveness of a chemodrug by acting as cytotoxic agent itself, if produced at significant concentrations, but also by inhibiting the ABC transporters responsible for MDR, when generated in non-toxic amount. Therefore, we considered it useful to perform some very preliminary biological experiments by using A375 melanoma cell line, overexpressing Pgp, MRP1, and BCRP (Figure 6), i.e., the main pumps effluxing DOX.

**Figure 7:**
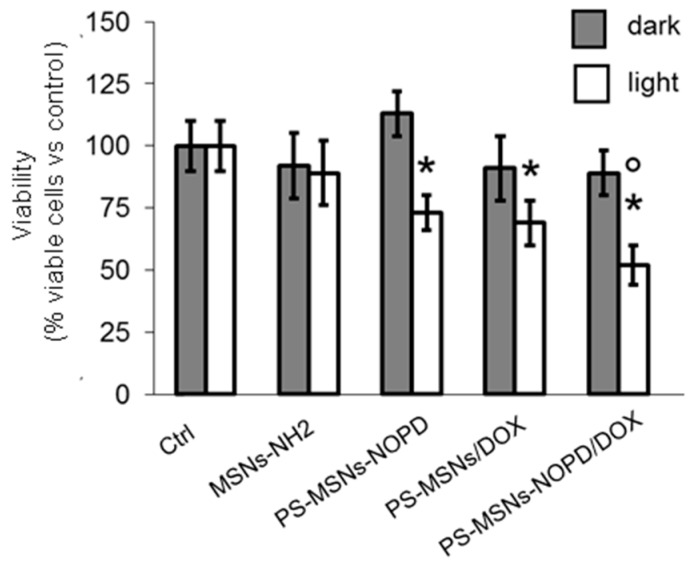
Cell viability of A375 cells, treated a reported in the Materials and Methods Section. Data are presented as means ± SD (n = 3 independent experiments). * *p* < 0.02: vs. Ctrl, irradiated cells (light); ° *p* < 0.05: PS-MSNs-NOPD/DOX vs. PS-MSNs-NOPD and PS-MSNs/DOX.

Thanks to the logical design of the MSNs, irradiation with blue light permits the selective excitation of the NOPD component, ruling out any participation of ^1^O_2_ (which would require green light excitation of the PS) in the cell viability. Figure 7 shows that the different samples of MSNs are well tolerated in the dark and that only a slight toxicity was observed upon blue light irradiation. Moreover, the very moderate phototoxicity observed in the sample with NOPD (PS-MSNs-NOPD) is basically the same to that of the sample, which does not integrate the NOPD (PS-MSNs/DOX), suggesting that under these experimental conditions NO is produced at not toxic doses.

Interestingly, irradiation of the whole nanoconstruct (PS-MSNs-NOPD/DOX) increases cytotoxicity. PS-MSNs-NOPD/DOX reduced cell viability more than PS-MSNs-NOPD and PS-MSNs/DOX, suggesting that the presence of NOPD enhances the efficacy of DOX. We believe that such effect can be tentatively attributed to the inhibition of the ABC transporters present on A375 cells by NO photogenerated, an event already observed in melanoma cells treated with free NOPD/DOX hybrids [25].

## 4. Conclusions

We have designed, synthesized and characterized a novel “three bullet” nanoconstruct based on mesoporous silica nanoparticles for potential combined cancer photo-chemotherapy. Such a multifunctional nanoplatform is able to generate ^1^O_2_ and NO under selective excitation with green and blue light, respectively, and release the noncovalently entrapped anticancer DOX under physiological conditions. A remarkable advantage of this system is the excellent preservation of the photochemical properties of the active components once simultaneously integrated in the same scaffold and despite the copresence of the DOX. This is the result of the strategy adopted which permitted to integrate the PS and the NOPD in the outer and the inner sites of the nanoscaffold, avoiding potential and undesired intermolecular photoprocesses (i.e., energy/electron transfer) which would have otherwise precluded the final goal. This allows the individual photoactivable components to be operated either singularly or in tandem upon the appropriate choice of the excitation wavelengths. At this regard, preliminary biological results performed on A375 cells expressing ABC transporters show a potentiated activity of DOX incorporated in our “three bullet” nanoconstruct upon irradiation with blue light, due to the effect of the NO photoreleased. Note that the reduction of the cell viability observed under light excitation (ca. 50%) is considered effective in overcoming DOX-resistance in cells expressing ABC transporters [47,48], as those used in the present work. Besides its efficacy, a plus of our approach is the absence of toxicity exerted by PS-MSNs/NOPD/DOX in the absence of irradiation. Since light can be easily controlled and directed towards tumor tissue, in a translational perspective, our approach can be considered a tumor-selective strategy that avoids undesired toxicity on nontransformed tissues and overcomes a severe limitation of currently used chemotherapy. Detailed photobiological investigations addressed to elucidate the single and the combined effects of all the active species produced by this novel multifunctional drug delivery system are currently under investigation in our laboratories.
